# Filling Defect of Ipsilateral Transverse Sinus in Acute Large Artery Occlusion

**DOI:** 10.3389/fneur.2022.863460

**Published:** 2022-05-10

**Authors:** Yi Chen, Sheng Zhang, Shenqiang Yan, Meixia Zhang, Ruiting Zhang, Feina Shi, David S. Liebeskind, Mark Parsons, Min Lou

**Affiliations:** ^1^Department of Neurology, The Second Affiliated Hospital of Zhejiang University, Hangzhou, China; ^2^Department of Neurology, Zhejiang Provincial People's Hospital, People's Hospital of Hangzhou Medical College, Hangzhou, China; ^3^Department of Neurology, Jinhua Municipal Central Hospital, Jinhua Hospital of Zhejiang University, Jinhua, China; ^4^Department of Radiology, The Second Affiliated Hospital of Zhejiang University, Hangzhou, China; ^5^UCLA Stroke Center, University of California, Los Angeles, Los Angeles, CA, United States; ^6^Department of Neurology, John Hunter Hospital, University of Newcastle, New Lambton Heights, NSW, Australia

**Keywords:** large artery occlusion in anterior circulation, transverse sinus, computer tomography, brain edema, reperfusion therapy

## Abstract

**Background and Purpose:**

Cerebral venous systems play a key role in regulating stroke outcomes. We aimed to elucidate the effect of the transverse sinus (TS) filling patterns on edema expansion and neurological outcomes in patients with acute large artery occlusion (LAO).

**Materials and Methods:**

We recruited consecutive patients with acute M1 middle cerebral artery and/or internal carotid artery occlusion who underwent pretreatment computed tomographic perfusion (CTP). On the reconstructed 4-dimensional computed tomographic angiography derived from CTP, the filling defect of the ipsilateral transverse sinus (FDITS) was defined as the length of contrast filling defect occupying at least half of the ipsilateral TS. An unfavorable outcome was defined as having a modified Rankin Scale (mRS) score of 3–6 at 3 months.

**Results:**

A total of 318 patients were enrolled in the final analysis and 70 (22.0%) patients had baseline FDITS. The presence of FDITS was associated with the baseline NIHSS (odds ratio [*OR*] 1.119; 95% *CI*, 1.051–1.192; *p* < 0.001) and poor arterial collaterals (*OR* 3.665; 95% *CI* 1.730–7.766; *p* = 0.001). In addition, FDITS was associated with 24-h brain edema expansion (*OR* 7.188; 95% *CI*, 3.095–16.696; *p* < 0.001) and 3-month unfavorable outcome (*OR* 8.143; 95% *CI* 2.547–26.041; *p* < 0.001) independent of arterial collateral status. In the subgroup analysis of patients with FDITS who received reperfusion therapy, no significant difference was found in the rate of edema expansion and unfavorable outcome between non-reperfusion and reperfusion subgroups (both *p* > 0.05).

**Conclusion:**

Filling defect of the ipsilateral transverse sinus was associated with edema expansion and an unfavorable outcome irrespective of the baseline arterial collateral status in patients with acute LAO, indicating that FDITS may be an important stroke-related prognostic imaging marker.

## Introduction

Despite the development of reperfusion therapy, the rate of severe disability and mortality in patients with acute large artery occlusion (LAO) remains high (1). Previous studies on acute ischemic stroke (AIS) have focused on arterial pathophysiology; however, it was recently discovered that cerebral venous systems also have a role in determining the prognosis of AIS after reperfusion therapy (2–4).

Cerebral veins have been used to predict stroke outcomes, though the exact mechanisms are still unclear (2, 3, 5–7). Cortical and deep veins were both suggested to be good venous markers for stroke outcomes (2, 3, 5–7). Nevertheless, in terms of patients with LAO, these veins may not reflect the entire venous drainage abnormalities in the corresponding regions supplied by cerebral arteries. Additionally, there were difficulties in recognizing the cortical and deep veins due to the broad variation of cerebral venous across different centers (8).

The transverse sinus (TS), functioning as a pool gathering cortical and deep veins, is superficial and easier to identify than cerebral veins (8). Additionally, the vein of Labbé collects the blood flow of the lateral temporal area supplied by the middle cerebral artery (MCA), joining into ipsilateral TS directly (9). Therefore, TS might be an appropriate target to reflect the abnormality of hemispheric venous drainage, especially in patients with acute LAO of the anterior circulation.

We thus evaluated the drainage pattern of TS on the reconstructed four-dimensional CT angiography (4D-CTA) derived from CT perfusion (CTP) in AIS patients with anterior circulation LAO and investigated whether the drainage abnormality of ipsilateral TS would influence their clinical outcomes.

## Subjects And Methods

### Ethics Statement

Each subject or an appropriate family member had given written informed consent prior to the study, and the protocols had been approved by the local ethics committee. All clinical investigations were conducted according to the principles expressed in the Declaration of Helsinki.

### Study Subjects

We retrospectively reviewed our prospectively collected database, namely, Comparison Influence to Prognosis of CTP and MRP in AIS Patients (CIPPIS, http://www.clinicaltrials.gov, Unique identifier: NCT03367286), of patients with consecutive AIS who were admitted within 9 h of stroke onset or with an unknown time of onset and underwent CTP before the treatment between January 2014 and January 2019. Reperfusion therapy, including intravenous thrombolysis and/or endovascular therapy, was given to eligible patients with informed consent according to the guidelines of the time. We enrolled patients who had middle cerebral artery M1 segment and/or intracranial internal carotid artery (ICA) occlusion on pretreatment 4D-CTA reconstructed from CTP. We excluded patients who (1) had pre-stroke a modified Rankin Scale score (mRS) >2; (2) had bilateral acute ischemic lesions; (3) had poor image quality due to severe head motion artifact on the image; and (4) could not tolerate follow-up CT/MRI or were lost to follow-up within 3 months.

Patients' demographic, clinical, laboratory, and radiological data, including age, sex, comorbid conditions, such as the history of stroke/transient ischemic attacks (TIA), hypertension, diabetes mellitus, coronary artery disease, and atrial fibrillation, were retrieved. Baseline neurologic severity was assessed by the National Institutes of Health Stroke Scale (NIHSS). Favorable outcome and unfavorable outcome were defined as mRS scores of 0–2 and 3–6 at 3 months, respectively (10).

### Imaging Protocols

All patients underwent baseline CTP, including non-contrast CT (NCCT) and volume perfusion CT (VPCT), and follow-up CTP or magnetic resonance perfusion or NCCT at 24-h after admission in accordance with our routine stroke imaging protocol (11). The detailed parameters are listed in the Supplementary Material. VPCT images were reconstructed to obtain time to maximum (Tmax) maps and 4D-CTA images were presented in axial, coronal, and sagittal planes with 20-mm-thick maximum intensity projections by commercial software (MIStar; Apollo Medical Imaging Technology, Melbourne, Australia).

### Defining the Filling Defect of The Ipsilateral Transverse Sinus (FDITS)

The contrast filling of TS of each hemisphere was evaluated on a coronal view of temporally fused maximum intensity projections (tMIPs) reconstructed from 4D-CTA images. According to the contrast filling pattern of the ipsilateral TS, the presence of a filling defect of the ipsilateral transverse sinus (FDITS) was confirmed by two steps: (1) contrast filling defect was defined as the width of the contrast filling in ipsilateral TS being ≤ 50% width of the contralateral TS (12); and (2) the length of contrast filling defect occupied at least half of the ipsilateral TS. Hypoplasia and aplasia of TS of the ischemic side were also identified as FDITS. A similar definition was used for the filling defect of contralateral TS (FDCTS) and all others were defined as symmetric TS. Both FDCTS and symmetric TS were set as non-FDITS.

### Assessment of Arterial Collaterals on 4D-CTA

Arterial collaterals were evaluated on peak arterial opacification (peak phase) and tMIPs were reconstructed from 4D-CTA images, and the regional leptomeningeal collateral (rLMC) score was graded on peak phase (rLMC-P) and tMIP (rLMC-M), respectively. The integrated collateral grading scale (CGS) was used to assess the arterial collateral status: poor collaterals (score 0: rLMC-P ≤ 11 and rLMC-M ≤ 16), intermediate collaterals (score 1: rLMC-P ≤ 11 and rLMC-M >16, or rLMC-P >11 and rLMC-M ≤ 16), and good collaterals (score 2: rLMC-P >11 and rLMC-M >16) (13).

### Defining Hypoperfusion and Ischemic Core

A threshold of Tmax > 6 s was used for the volumetric measurement of baseline and 24-h hypoperfusion areas (14). Baseline relative cerebral blood flow (rCBF) <30% was used for calculating the ischemic core volume (15). At 24 h, Diffusion Weighted Imaging (DWI) or NCCT was used to calculate the final infarct volume (13).

### Reperfusion, Hemorrhagic Transformation, and Brain Edema

Reperfusion ratio (RR) = (baseline hypoperfusion volume – 24-h hypoperfusion volume)/baseline hypoperfusion volume. Based on RR, we defined reperfusion as RR ≥80% and non-reperfusion as RR <80% (16).

Hemorrhagic transformation, including parenchymal hemorrhage (PH), was classified according to the European Cooperative Acute Stroke Study criteria. Symptomatic intracranial hemorrhage (sICH) was defined as any intracranial hemorrhage associated with an increase of ≥4 points on NIHSS or leading to death (17).

Brain edema was assessed with a 7-point scale at baseline NCCT and 24-h NCCT or DWI (18, 19). To minimize the grading error, 24-h brain edema expansion was defined as an increase of ≥2 in grade from baseline imaging to 24-h imaging (19).

### Reproducible Parameters

Two evaluators (Y.C. and S.Z.) who jointly evaluated the TS were blinded to the patients' 24-h imaging and clinical data. A single trained observer (Y.C.) measured the TS of all patients two times, at an interval of 3 months apart. Another observer (S.Z.) independently made the same evaluation. Similarly, rLMC, brain edema, and PH were assessed by two neurologists independently (Y.C and S.Z), respectively. The Kappa statistic was used to test the inter- and intra-rater reliability for detecting the presence of FDITS and PH, and the weighted Kappa statistic was used to test the inter- and intra-rater reliability for rLMC and brain edema score.

There was a good level of inter- and intra-observer agreement in the evaluation of arterial collaterals status (weighted κ = 0.80 and 0.86), FDITS (κ = 0.81 and 0.92), and an excellent level of agreement for brain edema (weighted κ = 0.86 and 0.90), hemorrhagic transformation (κ = 0.86 and 0.99).

### Statistical Analysis

All numeric variables were expressed as mean ± SD and median (interquartile range, IQR). Categorical variables were presented as frequency (percentage). Fisher's exact test was used to compare the dichotomous variables between groups, while Mann–Whitney *U*-test was used for ordered categorical variables, and independent samples 2-tailed *t-*test or Mann–Whitney *U-*test was used for continuous variables, depending on the normality of the distribution. Variables identified by univariate analysis (*p* < 0.05) were included in the binary logistic regression model except for potential linearly correlated ones. Independent factors for the presence of FDITS, edema expansion, and unfavorable outcomes were evaluated using the binary logistic regression analysis, respectively. Then, the subgroup analysis was made for the effects of FDITS on edema expansion and unfavorable outcomes under the condition of different collateral status and reperfusion status, separately. All analyses were performed blinded to the participant identifying information. Statistical significance was set as a probability value of <0.05. The statistical analysis was performed using SPSS 18 (SPSS Inc., Chicago, IL, USA).

## Results

### Overall Characteristics

**Figure 1:**
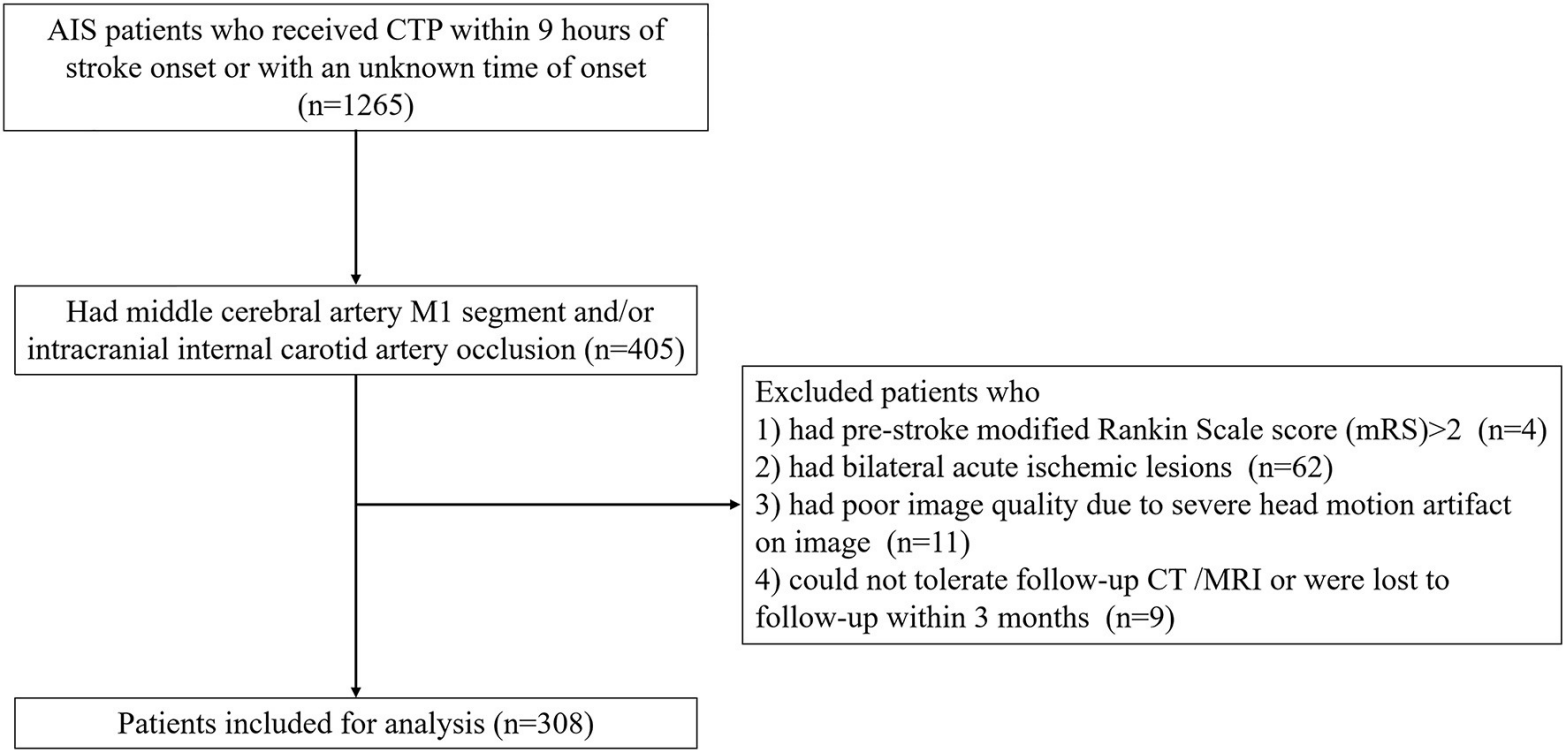
The flowchart of inclusion and exclusion.

A total of 318 patients were enrolled in the final analysis (Figure 1). The average age was 70 ± 13 years and 197 (61.9%) patients were men. The median baseline NIHSS was 14 (IQR 10–18). Among them, 287 (90.3%) patients received reperfusion therapy, including endovascular treatment (*n* = 142). FDITS occurred in 70 (22.0%) patients. Non-FDITS was identified in 248 (78.0%) patients, including 82 FDCTS and 166 symmetric TS. Baseline poor arterial collaterals (score 0) were found in 168 (54.9%) patients. At 24 h, 155 (48.7%) patients underwent brain edema expansion. At 3 months, 197 (61.9%) patients suffered unfavorable outcomes.

### Association Between FDITS and Baseline Characteristics

Table 1 shows the baseline characteristics and outcomes dichotomized by the presence of FDITS. Compared with the non-FDITS group, patients with FDITS had higher baseline NIHSS, larger baseline hypoperfusion volume and ischemic core volume, and a higher rate of poor arterial collaterals.

**Table 1:** Baseline characteristics and outcomes dichotomized by the presence of FDITS.

	**FDITS**	**Non-FDITS**	***P*-value**
	**(*n* = 70)**	**(*n* = 248)**	
**Demographics and clinical features**			
Age, year	71 ± 14	70 ± 13	0.684
Male, n (%)	43 (61.4)	154 (62.1)	0.999
Baseline NIHSS, median (IQR)	18 (14–20)	13 (9–16)	<0.001^*^
Onset to door time, min^a^,	194 (120–318)	174 (86–260)	0.167^*^
median (IQR)			
Onset to needle time, min^b^,	259 (168–337)	208 (135–296)	0.061^*^
median (IQR)			
Baseline systolic BP, mmHg	155 ± 25	150 ± 23	0.167
Baseline diastolic BP, mmHg	84 ± 15	83 ± 14	0.444
Baseline blood glucose, mmol/L	7.31 ± 2.45	7.65 ± 2.40	0.310
INR, median (IQR)	1.04 (0.98–1.11)	1.02 (0.98–1.10)	0.246^*^
History of atrial fibrillation, *n* (%)	36 (52.2)	123 (50.4)	0.892
History of hypertension, *n* (%)	50 (72.5)	154 (63.1)	0.156
History of diabetes mellitus, *n* (%)	9 (13.0)	46 (18.9)	0.289
History of coronary artery disease, *n* (%)	8 (11.6)	33 (13.5)	0.840
History of stroke/ TIA, *n* (%)	13 (18.8)	55 (22.5)	0.620
**Baseline imaging data**			
Baseline hypoperfusion volume,	154 (97–213)	124 (74–168)	0.005^*^
ml, median (IQR)			
Baseline ischemic core volume,	80 (41–140)	48 (25-84)	<0.001^*^
ml, median (IQR)			
Poor collaterals, *n* (%)	57 (83.8)	111 (46.6)	<0.001
Baseline edema score, median (IQR)	0 (0–1)	0 (0–0)	0.021^*^
**Outcomes**			
PH, *n* (%)	12 (17.1)	25 (10.1)	0.137
sICH, *n* (%)	6 (8.6)	16 (6.5)	0.594
Edema score at 24-h, median (IQR)	3 (2–4)	1 (0–2)	<0.001^*^
Brain edema expansion, *n* (%)	62 (88.6)	93 (37.5)	<0.001
Reperfusion, *n* (%)^c^	21 (48.8)	111 (58.7)	0.306
Unfavorable outcome, *n* (%)	65 (92.9)	132 (53.2)	<0.001

* *Mann–Whitney U-test*.

a * Estimated as the midpoint of sleep (i.e., the time between going to sleep and waking up with symptoms) among patients with wake-up stroke*.

b * Evaluated in patients who received intravenous thrombolysis (n = 243, FDITS vs. non-FDITS = 50 vs. 193)*.

c * Evaluated in patients who had both baseline and 24-h perfusion images (n = 232, FDITS vs. non-FDITS = 43 vs. 189)*.

*FDITS, filling defect of the ipsilateral transverse sinus; NIHSS, National Institutes of Health Stroke Scale; BP, blood pressure; INR, International normalized ratio; TIA, Transient ischemic attack; PH, parenchymal hemorrhage; sICH, symptomatic intracranial hemorrhage*.

Binary logistic regression analysis revealed that the presence of FDITS was independently associated with baseline NIHSS (odds ratio [OR] 1.119; 95% *CI* 1.051–1.192; *p* < 0.001) and poor arterial collaterals (*OR* 3.665; 95% *CI* 1.730–7.766; *p* = 0.001) after adjusting for ischemic core volume (*OR* 1.002; 95% *CI* 0.997–1.007; *p* = 0.372).

### The Associations of FDITS With Edema Expansion and Unfavorable Outcome

The patients with FDITS showed a higher rate of brain edema expansion and unfavorable outcomes (Table 1). The univariate analysis for the association between factors and edema expansion and outcome is separately shown in Supplementary Tables 1, 2. The binary logistic regression analysis showed that the presence of FDITS was independently associated with unfavorable outcomes (92.9 vs. 53.2%, OR 8.143; 95% CI 2.547–26.041; p < 0.001) (Table 2). In addition, we found that the presence of FDITS was associated with edema expansion (88.6 vs. 37.5%, OR 7.188; 95% CI 3.095–16.696; p < 0.001) (Supplementary Table 3). Baseline hypoperfusion volume remarkably correlated linearly with the baseline ischemic volume (rs = 0.778, p < 0.001) and thus was not included in the binary logistic regression.

**Table 2:** Binary logistic regression analysis for unfavorable outcome.

	**OR**	**95% CI**	***P-*value**
Age, year	1.030	1.004–1.056	0.021
Male	0.464	0.235–0.918	0.027
History of hypertension	1.573	0.821–3.013	0.172
Onset to door time, per minute	1.003	1.001–1.005	0.012
Baseline NIHSS	1.068	1.001–1.140	0.045
Baseline ischemic core, per ml	1.011	1.002–1.020	0.018
FDITS	8.143	2.547–26.041	<0.001
Poor collaterals	3.115	1.575–6.162	0.001
Application of endovascular therapy	0.390	0.202–0.754	0.005

*FDITS, filling defect of the ipsilateral transverse sinus; NIHSS, National Institutes of Health Stroke Scale; OR, odds ratio; CI, confidence interval*.

### Subgroup Study in Patients With FDITS and Non-FDITS

**Figure 2:**
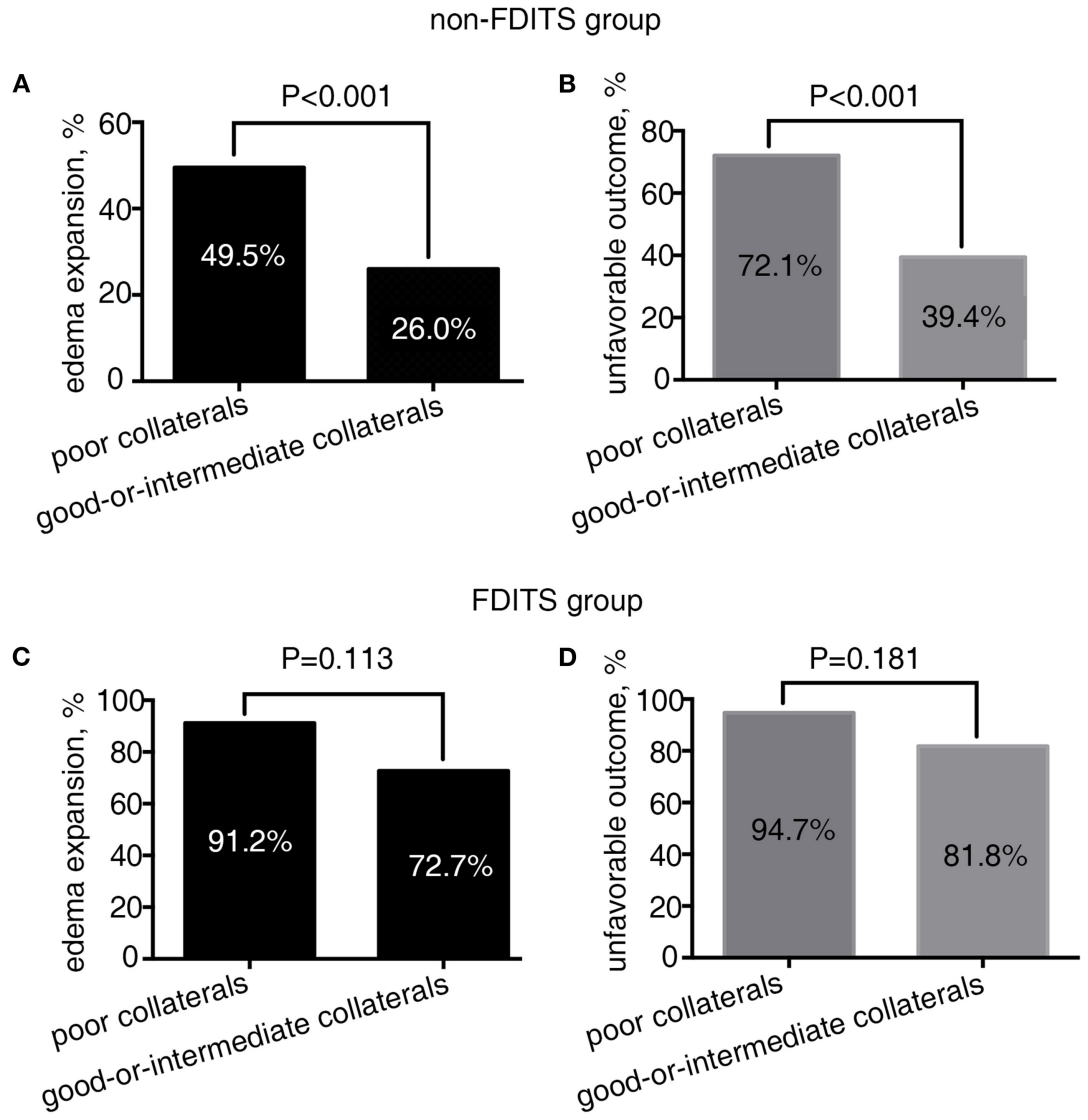
The relationship between collateral status and outcome in the filling defect of ipsilateral transverse sinus (FDITS) and non-FDITS groups. The poor collaterals subgroup was more likely to undergo edema expansion **(A)** and an unfavorable outcome **(B)** than those with good-or-intermediate collaterals in patients with non-FDITS. While in the FDITS group, no significant difference was found in the rate of edema expansion **(C)** or an unfavorable outcome **(D)** between patients with poor and good-or-intermediate collaterals.

As Figure 2 shows, in patients with FDITS, the rate of edema expansion (91.2 vs. 72.7%, *p* = 0.113) and unfavorable outcome (94.7 vs. 81.8%, *p* = 0.181) was comparable between the poor collaterals subgroup and the good-or-intermediate subgroup. However, among patients with non-FDITS, the poor collaterals subgroup was likely to have a higher rate of edema expansion (49.5 vs. 26.0%, *p* < 0.001) and unfavorable outcome (72.1 vs. 39.4%, *p* < 0.001) than the good-or-intermediate collaterals subgroup.

**Figure 3:**
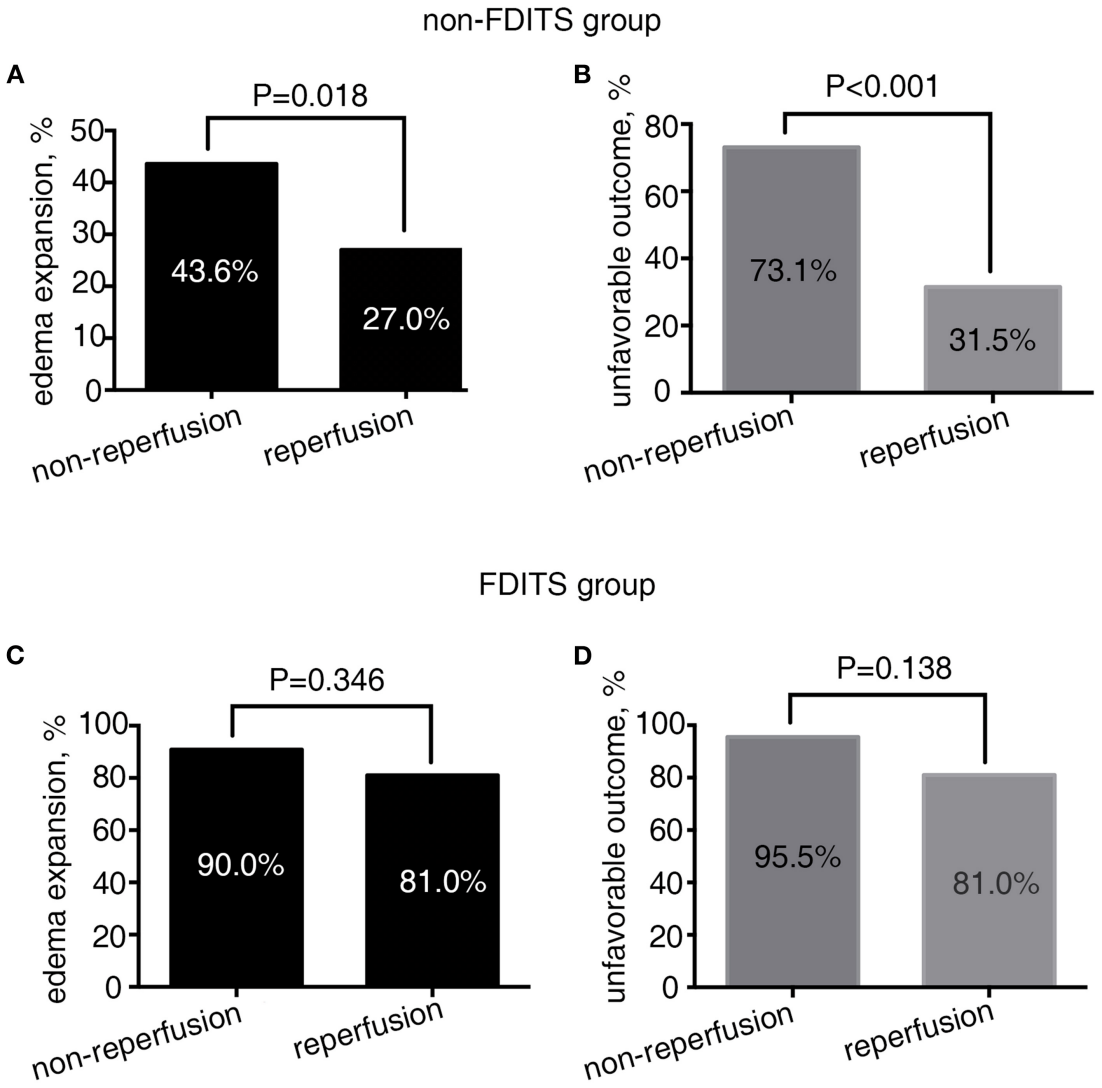
The relationship between reperfusion status and outcome in the FDITS and non-FDITS groups. A non-reperfusion subgroup was more likely to undergo edema expansion **(A)** and unfavorable outcome **(B)** than reperfusion subgroup in patients with non-FDITS. While in the FDITS group, no significant difference was found in the rate of edema expansion **(C)** or unfavorable outcome **(D)** between two subgroups.

As Figure 3 shows, in patients with FDITS, no significant difference was found in the rate of edema expansion (90.9 vs. 81.0%, *p* = 0.346) or unfavorable outcome (95.5 vs. 81.0%, *p* = 0.138) between the non-reperfusion subgroup and the reperfusion subgroup. However, patients with non-FDITS were likely to develop a higher rate of edema expansion (43.6 vs. 27.0%, *p* = 0.018) and unfavorable outcome (73.1 vs. 31.5%, *p* < 0.001) when reperfusion was not achieved.

**Figure 4:**
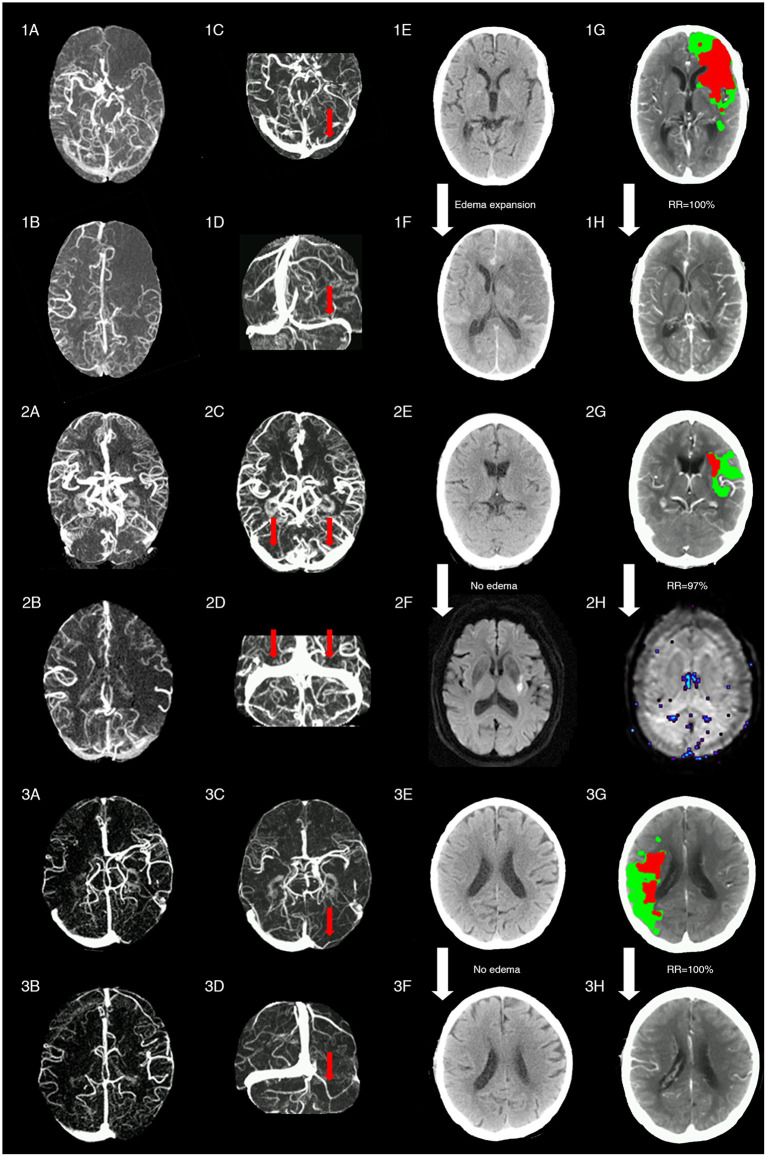
Representative images of patients with different status of ipsilateral transverse sinus and collaterals. Patient 1 with acute left middle cerebral artery occlusion (MCAO) (baseline NIHSS = 14) presented with poor collaterals [integrated collateral grading scale (CGS) =0] (1A,1B) and FDITS (the red arrow in 1C,1D) on 4-dimensional computed tomographic angiography (4D-CTA). The patient received intravenous thrombolysis bridging with endovascular therapy at 2.5 h from stroke onset. At 24 h after reperfusion therapy, this patient developed brain edema expansion on non-contrast CT (NCCT) from baseline score 0 (1E) to 2 (1F), although reperfusion rate was 100% with hypoperfusion (red plus green area) volume changing from 220 ml (1G) to 0 ml (1H) on lesion map. The modified Rankin scale (mRS) score was 4 at 3 months. Patient 2 with acute left MCAO (baseline NIHSS = 13), presented with good collaterals (CGS = 2) (2A,2B) and symmetric bilateral transverse sinuses (non-FDITS) (two red arrows in 2C,2D) on 4D-CTA. The patient received intravenous thrombolysis at 4 h from stroke onset. At 24 h after reperfusion therapy, this patient developed no brain edema with edema score keeping 0 from baseline NCCT (2E) to 24-h diffusing weighted imaging (DWI) (2F), and reperfusion rate was 97% with hypoperfusion (red plus green area) volume changing from 29 ml (2G) to 1 ml (2H) on lesion map. The mRS score was 0 at 3 months. Patient 3 with acute right MCAO (baseline NIHSS = 11), presented with good collaterals (CGS = 2) (3A,3B) and filling defect of contralateral transverse sinus (FDCTS) (the red arrow in 3C,3D) on 4D-CTA. The patient received intravenous thrombolysis bridging with endovascular therapy at 2 h from stroke onset. After reperfusion therapy, this patient developed no brain edema with edema score keeping 0 on NCCT from baseline (3E) to 24 h (3F), and reperfusion rate was 100% with hypoperfusion (red plus green area) volume changing from 137 ml (3G) to 0 ml (3H) on lesion map. The mRS score was 0 at 3 months.

Figure 4 shows three cases with representative images.

## Discussion

In our study, we found that FDITS had a close relationship with baseline arterial collateral status, and FDITS was associated with 24-h edema expansion and subsequently 3-month unfavorable outcome independent of the baseline arterial collateral status.

The venous drainage system is a major blood reservoir. A prior study revealed that the ipsilateral absent filling of the superficial middle cerebral vein (SMCV) was influenced by the severity of reduction in upstream arterial flow among patients with stroke (2). Considering the high rate of congenital hypoplastic TS, it is difficult to clarify whether the asymmetry of TS was congenital or a result of ischemia in patients with stroke in our study since we had no pre-stroke imaging of the same patient for comparison. However, we infer that stroke itself may influence TS symmetry because the rate of symmetric TS was actually changed in patients with stroke. In a healthy population, 50–60% of patients had right dominant TS and 10–20% had left dominant TS, leaving the occurrence rate of symmetric TS as low as 30% (20, 21), whereas in other two stroke studies, 57% of patients had symmetric TS and 21–30% had “hypoplasia” or “occlusion” of ipsilateral TS (22, 23). Similarly, in our study, 52.2% of patients presented symmetric TS and 22% presented FDITS, indicating that TS morphology may be modulated by ischemic stroke and might be a stroke-related imaging feature.

In addition, our findings on the relationship between disturbed drainage of TS and poor arterial collaterals suggest that venous outflow is influenced by the severity of low arterial flow. This finding is consistent with a previous study which demonstrated that the patients with asymmetrical clearance of TS were more likely to have incomplete arterial collateral filling on dynamic magnetic resonance angiography (*p* = 0.015) (24). A similar phenomenon was reported in the MCA occlusion model of the African green monkey, showing that the poor collateral circulation was related to a back and forth blood pooling with no venous return into cortical veins, leading to no flow into the venous sinus (25).

Previous studies described the independent impact of venous drainage patterns on outcomes in patients with AIS (2, 3), which supported the view that cerebral veins played a vital role in the maintenance of CBF and brain function after ischemia. In the current study, we first identified that FDITS was also independently associated with unfavorable outcomes in the acute LAO of anterior circulation. Importantly, we found that although FDITS and poor collaterals were both independently associated with 24-h brain edema expansion, their prognostic values were different. When FDITS was absent, about 50% of patients who had poor collaterals suffered severe brain edema. However, if baseline FDITS was present, the possibility of an obvious edema expansion within 24 h was as high as about 90% regardless of the arterial collateral status. Decreased cerebral venous outflow in ipsilateral TS would greatly change the venous pressure, which was previously suggested as the primary driving force in the development of brain edema. When venous pressure is increased beyond the tissue pressure, the pressure difference might interfere with venous reflux and lead to parenchymal edema and subsequent unfavorable clinical outcome (2, 26, 27). Actually, ischemic brain edema is a crucial cause of death for stroke survivors (28), as malignant cerebral infarct, usually related to poor collaterals, leads to a poor outcome. It is thus worth exploring in future studies whether the early identification of FDITS at admission in patients with acute LAO would help to guide appropriate treatment to prevent or alleviate edema expansion by all means, for example, the widely announced early decompressive hemicraniectomy (28), or intravenous glyburide as a promising intervention (29), in addition to reperfusion effort (30).

However, the subgroup analysis result is surprising because patients with FDITS still had edema expansion at 24 h and an unfavorable outcome at 3 months even though they were treated with reperfusion therapy and even if reperfusion was obtained. This is different from the previous result, which revealed no significant difference in outcome between patients with or without ipsilateral SMCV if reperfusion was successfully achieved (2). It is believed that cerebral veins have sufficient anastomosis and the drainage area of TS is obviously much larger than that of cortical veins. Thus, it might be assumed that the occurrence of FDITS indicates the possibility of venous collateral failure to severe arterial ischemia, which would not be compensated even after successful reperfusion, leading to a synergistic influence on the tissue outcome (8). Although we could not jump to doubt whether reperfusion therapy might be of limited significance in patients with initial FDITS according to the current small sample subgroup results, our study emphasizes the need for ancillary judgment of venous drainage to guide reperfusion therapy, and future large prospective studies are needed to clarify this issue.

Our study has limitations attributed to its retrospective collection of data in a single center with a moderate sample size, which may pose a potential risk of selection bias. Second, the edema evaluation scale may not well differentiate vasogenic edema from infarction. To minimize the impact of this error, we focused on 24-h edema change to reflect relatively actual edema development. Third, due to the lack of a gold standard, such as DSA, the accuracy of 4D-CTA in identifying the filling defect of transverse sinus needs to be further validated. Fourth, we could not differentiate the asymmetry of TS as nature or nurture from baseline 4D-CTA due to its recognition ability of jugular foramen dominancy. Fifth, 4D-CTA derived from CTP would not necessarily be performed on patients with LAO who arrive early at some large centers, which would further limit utility. In addition, we did not analyze the drainage pattern changes of TS after reperfusion therapy due to the lack of enough follow-up image data. Further studies are still needed to prove our findings and investigate the possible mechanisms.

## Conclusion

The filling defect of the ipsilateral transverse sinus was strongly associated with the level of arterial collateral flow. It was associated with edema expansion and an unfavorable outcome, irrespective of baseline arterial collaterals. FDITS might be identified as a potential imaging marker for neurological outcomes in acute LAO of the anterior circulation, and prevention of edema expansion might be an important ultra-early target once FDITS is identified.

## Data Availability Statement

The raw data supporting the conclusions of this article will be made available by the authors, without undue reservation.

## Ethics Statement

The studies involving human participants were reviewed and approved by the Ethics Committee of SAHZU. The patients/participants provided their written informed consent to participate in this study.

## Author Contributions

YC, SZ, and ML contributed to the conception and design of the study. YC, SZ, SY, MZ, FS, and MP contributed to the acquisition and analysis of the data. YC, SZ, DL, and ML contributed to drafting the text. YC, SZ, and RZ prepared the figures. ML is responsible for the overall content as guarantor. All authors contributed to the article and approved the submitted version.

## Conflict of Interest

The authors declare that the research was conducted in the absence of any commercial or financial relationships that could be construed as a potential conflict of interest.

## Publisher's Note

All claims expressed in this article are solely those of the authors and do not necessarily represent those of their affiliated organizations, or those of the publisher, the editors and the reviewers. Any product that may be evaluated in this article, or claim that may be made by its manufacturer, is not guaranteed or endorsed by the publisher.
