# Supplementary material for: Filling Defect of Ipsilateral Transverse Sinus in Acute Large Artery Occlusion
Source: Front Neurol. 2022 May 10;13:863460. doi: 10.3389/fneur.2022.863460 (PMC9127321; doi:10.3389/fneur.2022.863460)
Supplement: Supplementary file 1 [file Data_Sheet_1.docx]

**Supplementary material**

The detailed CT and MR parameters

The effective dose (calculated by multiplying dose-length products with published conversion factors) amounted to 3.68 mSV for VPCT and 2.19 mSV for NCCT acquisition. Magnetic resonance perfusion (MRP) was performed on a 3.0-T system (Sigma Excite HD, General Electric, Milwaukee, USA). Foam pads were inserted into the space between the patient’s head and the MRI head coil to minimize head motion. The MRP protocol included PWI, which was performed with gradient echo-planar imaging (field of view=240mm,TR=1500ms,TE=30ms, acquisition matrix = 128 × 128, repetitive scanning times = 50, gadolinium dose = 15 mL, contrast speed = 4–5 mL/s, duration = 1 min 15 s). Other sequences included DWI (TR = 4000 ms; TE = 69.3 ms; b-value = 1000 s/mm2; slice thickness 5.0 mm; inter-slice gap P = 1.0 mm), time-of-flight MRA (TR = 20 ms; TE = 3.2 ms; flip angle = 15°; slice thickness = 1.4 mm, three slabs), and SWI (used 11 equally spaced echoes: TE = 4.5 ms (first echo); inter-echo spacing 4.5 ms; TR = 58 ms; flip angle 20°; slice thickness 2.0 mm with no gap between slices).
