# Supplementary material for: Filling Defect of Ipsilateral Transverse Sinus in Acute Large Artery Occlusion
Source: Front Neurol. 2022 May 10;13:863460. doi: 10.3389/fneur.2022.863460 (PMC9127321; doi:10.3389/fneur.2022.863460)
Supplement: Supplementary file 2 [file Data_Sheet_2.docx]

**Supplementary Table 1**

**Baseline characteristics dichotomized with brain edema expansion**

| Characteristics | Edema expansion  (n=155) | No edema expansion  (n=163) | *P* value |
| --- | --- | --- | --- |
| Demographics and clinical features | | | |
| Age, year | 71±12 | 69±14 | 0.346 |
| Male, n (%) | 91 (58.7) | 106 (65.0) | 0.251 |
| Baseline NIHSS, median (IQR) | 15 (12-19) | 12 (7-16) | <0.001^*^ |
| Onset to door time, min^a^, median (IQR) | 190 (118-294) | 160 (75-255) | 0.046^*^ |
| Onset to needle time, min^b^, median (IQR) | 239 (155-328) | 194 (135-277) | 0.013^*^ |
| Baseline systolic BP, mmHg | 152±26 | 150±21 | 0.362 |
| Baseline diastolic BP, mmHg | 83±15 | 83±14 | 0.928 |
| Baseline blood glucose, mmol/L | 7.55±2.20 | 7.59±2.60 | 0.883 |
| INR, median (IQR) | 1.03 (0.98-1.10) | 1.01(0.98-1.09) | 0.170^*^ |
| History of atrial fibrillation, n (%) | 86 (56.6) | 73 (45.3) | 0.055 |
| History of hypertension, n (%) | 102 (67.1) | 102 (63.4) | 0.553 |
| History of diabetes mellitus, n (%) | 29 (19.1) | 26 (16.1) | 0.553 |
| History of coronary artery disease, n (%) | 23 (15.1) | 18 (11.2) | 0.319 |
| History of stroke/ TIA, n (%) | 32 (21.1) | 26 (22.4) | 0.786 |
| Baseline imaging data | | | |
| Baseline hypoperfusion volume, ml, median (IQR) | 143 (100-206) | 107 (59 -163) | <0.001^*^ |
| Baseline ischemic core volume, ml, median (IQR) | 80 (43-131) | 36 (30-65) | <0.001^*^ |
| Poor collaterals, n (%) | 107 (72.3) | 61 (38.6) | <0.001 |
| FDITS, n (%) | 62 (40.0) | 8 (4.9) | <0.001 |
| Reperfusion therapy, n (%) | 138 (89.0) | 149 (91.4) | 0.571 |
| Application of endovascular therapy, n (%) | 71 (45.8) | 71(43.6) | 0.735 |

^*^Mann- Whitney U test

^a^ Estimated as the midpoint of sleep (i.e. the time between going to sleep and waking up with symptoms) among wake-up stroke patients.

^b^ Evaluated in patients who received intravenous thrombolysis (n=243, 111 of edema expansion vs. 132 of no edema expansion).

NIHSS, National Institutes of Health Stroke Scale; BP, blood pressure; INR, International normalized ratio; TIA, Transient ischemic attack; FDITS, filling defect of ipsilateral transverse sinus.

**Supplementary Table 2**

**Baseline characteristics and image outcomes dichotomized with favorable and unfavorable outcome**

| Characteristics | Favorable outcome  (n=121) | Unfavorable outcome  (n=197) | *P* value |
| --- | --- | --- | --- |
| Demographics and clinical features | | | |
| Age, year | 66±15 | 73±12 | <0.001 |
| Male, n (%) | 85 (70.2) | 112 (56.9) | 0.018 |
| Baseline NIHSS, median (IQR) | 10 (6-15) | 15 (12-18) | <0.001^*^ |
| Onset to door time, min^a^, median (IQR) | 151 (81-240) | 185 (102-294) | 0.034^*^ |
| Onset to needle time, min^b^, median (IQR) | 201 (131-281) | 227 (143-316) | 0.137^*^ |
| Baseline systolic BP, mmHg | 147±22 | 154±24 | 0.010 |
| Baseline diastolic BP, mmHg | 84±14 | 83±15 | 0.808 |
| Baseline blood glucose, mmol/L | 7.54±2.65 | 7.49±2.26 | 0.869 |
| INR, median (IQR) | 1.01 (0.97-1.07) | 1.03 (0.98-1.11) | 0.058^*^ |
| History of atrial fibrillation, n (%) | 55 (46.6) | 104 (53.3) | 0.294 |
| History of hypertension, n (%) | 68 (57.6) | 136 (69.7) | 0.037 |
| History of diabetes mellitus, n (%) | 19 (16.1) | 36 (18.5) | 0.648 |
| History of coronary artery disease, n (%) | 11 (9.3) | 30 (15.4) | 0.166 |
| History of stroke/ TIA, n (%) | 23 (19.5) | 45 (23.1) | 0.483 |
| Baseline imaging data | | | |
| Baseline hypoperfusion volume, ml, median (IQR) | 103 (55-144) | 146 (94-205) | <0.001^*^ |
| Baseline ischemic core volume, ml, median (IQR) | 35 (18-60) | 73 (36-127) | <0.001^*^ |
| Poor collaterals, n (%) | 34 (30.1) | 134 (69.4) | <0.001 |
| FDITS, n (%) | 5 (4.1) | 65 (33.0) | <0.001 |
| Reperfusion therapy, n (%) | 116 (95.9) | 171 (86.8) | 0.010 |
| Application of endovascular therapy, n (%) | 63 (52.1) | 79 (40.1) | 0.048 |
| Image outcomes at 24 hours | | | |
| PH, n (%) | 5 (4.1) | 32 (16.4) | 0.001 |
| sICH, n (%) | 3 (2.5) | 19 (9.6) | 0.021 |
| Brain edema expansion, n (%) | 23 (19.0) | 132 (67.0) | <0.001 |
| Reperfusion, n (%)^c^ | 80 (78.4) | 52 (40.0) | <0.001 |

^*^Mann- Whitney U test

^a^ Estimated as the midpoint of sleep (i.e. the time between going to sleep and waking up with symptoms) among wake-up stroke patients.

^b^ Evaluated in patients who received intravenous thrombolysis (n=243, 101 of favorable outcome vs. 142 of unfavorable outcome)

^c^ Evaluated in patients with baseline and 24-hours perfusion data (n=232, 102 of favorable outcome vs. 130 of unfavorable outcome).

NIHSS, National Institutes of Health Stroke Scale; BP, blood pressure; INR, International normalized ratio; TIA, Transient ischemic attack; FDITS, filling defect of ipsilateral transverse sinus; PH, parenchymal hemorrhage; sICH, symptomatic intracranial hemorrhage.

**Supplementary Table 3**

**Binary logistic regression analysis for 24-hour brain edema expansion**

|  | OR | 95% CI | *P* value |
| --- | --- | --- | --- |
| Onset to door time, per minute | 1.001 | 0.999-1.002 | 0.416 |
| Baseline NIHSS | 1.083 | 1.022-1.147 | 0.007 |
| Baseline ischemic core volume, per ml | 1.009 | 1.003-1.015 | 0.006 |
| FDITS | 7.188 | 3.095-16.696 | <0.001 |
| Poor collaterals | 1.837 | 1.006-3.353 | 0.048 |

NIHSS, National Institutes of Health Stroke Scale; FDITS, filling defect of ipsilateral transverse sinus; OR, odds ratio; CI, confidence interval.
